# Ferulic Acid-Loaded Polymeric Nanoparticles for Potential Ocular Delivery

**DOI:** 10.3390/pharmaceutics13050687

**Published:** 2021-05-11

**Authors:** Alessia Romeo, Teresa Musumeci, Claudia Carbone, Angela Bonaccorso, Simona Corvo, Gabriella Lupo, Carmelina Daniela Anfuso, Giovanni Puglisi, Rosario Pignatello

**Affiliations:** 1 PhD in Nurosciences, Department of Drug and Health Sciences, University of Catania, viale A. Doria 6, 95125 Catania, Italy; alessia.romeo@phd.unict.it (A.R.); ccarbone@unict.it (C.C.); abonaccorso@unict.it (A.B.); simona.corvo09@gmail.com (S.C.); puglisig@unict.it (G.P.); r.pignatello@unict.it (R.P.); 2 NANO-i, Research Centre for Ocular Nanotechnology, University of Catania, viale A. Doria 6, 95125 Catania, Italy; 3 Department of Biomedical and Biotechnological Sciences, University of Catania, 95127 Catania, Italy; daniela.anfuso@unict.it

**Keywords:** antioxidant, PLA, PLGA, retinal pericytes, endothelial cell, controlled release

## Abstract

Ferulic acid (FA) is an antioxidant compound that can prevent ROS-related diseases, but due to its poor solubility, therapeutic efficacy is limited. One strategy to improve the bioavailability is nanomedicine. In the following study, FA delivery through polymeric nanoparticles (NPs) consisting of polylactic acid (NPA) and poly(lactic-co-glycolic acid) (NPB) is proposed. To verify the absence of cytotoxicity of blank carriers, a preliminary in vitro assay was performed on retinal pericytes and endothelial cells. FA-loaded NPs were subjected to purification studies and the physico-hemical properties were analyzed by photon correlation spectroscopy. Encapsulation efficiency and in vitro release studies were assessed through high performance liquid chromatography. To maintain the integrity of the systems, nanoformulations were cryoprotected and freeze-dried. Morphology was evaluated by a scanning electron microscope. Physico-chemical stability of resuspended nanosystems was monitored during 28 days of storage at 5 °C. Thermal analysis and Fourier-transform infrared spectroscopy were performed to characterize drug state in the systems. Results showed homogeneous particle populations, a suitable mean size for ocular delivery, drug loading ranging from 64.86 to 75.16%, and a controlled release profile. The obtained systems could be promising carriers for ocular drug delivery, legitimating further studies on FA-loaded NPs to confirm efficacy and safety in vitro.

## 1. Introduction

Oxidative stress is able to involve morphological and functional alterations to retinal tissues, playing a key role in the onset and progression of retinal diseases, such as age-related macular degeneration (AMD), glaucoma, diabetic retinopathy (DR), and retinal vein occlusion (RVO) [1]. Recent clinical studies have demonstrated the potential health benefits obtained with the consumption of fruit and vegetables rich in phytochemicals, such as polyphenols, on visual function. Thanks to the pluri-pharmacological effects, these molecules might be able to slow down and prevent the progression of the aforementioned pathologies [2,3].

The most attractive polyphenol effects in these diseases are wielded on oxidative stress pathways, where they are able to suppress the harmful effect of the reactive oxygen species (ROS) [4]. For this reason, antioxidant molecules are gaining importance as a promising therapeutic strategy in treatment/prevention of eye chronic disease. A comparative study regarding the properties of various antioxidants including ascorbic acid, ferulic acid (FA), α-tocopherol and β-carotene, has shown that FA is the most efficient among the tested antioxidants [5,6]. Thanks to its phenolic nucleus and an extended side chain conjugation, this substance can act as a potent antioxidant in both isolated membranes and intact cells, because it is able to form a resonance-stabilized phenoxy radical, thereby inhibiting lipid peroxidation and ROS production.

FA (4-hydroxy-3-methoxycinnamic acid) is a phenolic compound and a notable biological and structural component of the plant cells. It is one of the most abundant phenolic acids in plants and might be found in high concentration in food such as whole grains (1–3 mg/100 g), fruits, and vegetables (800 mg/100 g) [7]. FA exhibits a wide spectrum of beneficial activity for human health, it was tested in vitro for its potential anti-inflammatory, anticancer, neuroprotective, anti-angiogenesis effects and was tested in vivo on mice for its antidiabetic, anticancer, antiapoptotic, and antioxidant properties [8]. Despite this, poor solubility of FA in aqueous solution remains a major limit for its bioavailability. In recent years, in order to overcome this problem and to improve the drug dissolution rate, many strategies were developed such as the drug complexation with hydroxypropyl-β-cyclodextrin (HP-β-CD), the inclusion in platforms composed of cocrystal, micelles, and nanogels, and the encapsulation in nanostructured lipid carriers (NLC) or chitosan NPs [9,10,11,12,13,14,15,16].

The use of biodegradable polymeric particles has been extensively studied to increase bioavailability, prolong controlled drug release, and avoid repeated ocular administration [17]. The use of polymeric NPs include many advantages, such as good control on size and size distribution, reduce clearance time, and protection and retention of the drug that improves bioavailability in intraocular or extraocular tissues [18]. Polymers frequently used to develop NPs for ocular delivery include poly(lactic acid) (PLA), poly(lactic acid)/poly(lactic-co-glycolic acid) (PLGA), polycaprolactone (PCL), and hyaluronic acid [19]. Despite this, to date, no study has been conducted on PLA or PLGA NPs for ocular delivery of FA. The use of PLA/PLGA carriers for ocular drug delivery (ODD) is sustained by their biocompatibility and biodegradability [18,20,21]. In a work by Gupta et al., sparfloxacin loaded in PLGA-NPs was administered to rabbits, showing to improve the residence time at the corneal surface with respect to conventional eye drops. In vivo studies of this formulation signalized that PLGA-NPs have a good stability and ocular tolerance. Moreover, in vivo degradation of PLGA mainly happens by hydrolysis, resulting in nontoxic lactic and glycolic acids, which enter to the tricarboxylic acid cycle to be metabolized in water, carbon dioxide, and energy [22]. A study conducted by Bourges et al. on PLA NPs showed that a single intravitreal injection in rats allows the system in retinal pigment epithelium (RPE) cells to be found, even after 4 months. Histology demonstrated the anatomical integrity of the injected eyes and the absence of toxic effects [17]. Administration by intravitreal injection has also been shown to be safe with PLA/PLGA microspheres, so the systems can be considered suitable for the treatment of diseases affecting the posterior segment of the eye [23]. In addition, several studies on polymeric nanoparticles (NPs) have used intravitreal injection as a route of administration, so the nanocarriers designed and discussed here could be used for this purpose [24,25,26,27].

The aim of this study was to prepare and characterize FA-PLA and PLGA NPs for potential ocular delivery, evaluating their physico-chemical, technological properties suitable for the selected site of administration, and a preliminary in vitro study was performed. The two unloaded nanocarriers were subjected to in vitro cell viability studies on primary endothelial cells and primary retinal pericytes to assess the absence of cytotoxicity. The two nanoformulations were loaded with the drug and investigated to determine the mean size, polydispersity index (PDI), zeta potential (ZP), pH, osmolarity, encapsulation efficiency (EE), and release profile until 48 h. Centrifugation and dialysis were carried out to eliminate both surfactant and the unloaded drug, and to select the most efficient purification method. The final formulations were cryoprotected and freeze-dried both to prevent premature drug release and to avoid hydrolysis of the polymeric material from the aqueous suspension. NPs morphology was assessed by SEM analysis. To evaluate the stability after resuspension, physico-chemical parameters were monitored during 28 days of storage at 5 °C. Freeze-dried samples were subjected to thermal analysis through differential scanning calorimetry (DSC) and FT-IR spectroscopy.

## 2. Materials and Methods

### 2.1. Materials

Trans-Ferulic acid, Resomer^®^ R 202 H, acid terminated, Mw 10.000–18.000 (PLA), Resomer^®^ RG 752 H, acid terminated, lactide:glycolide 75:25, Mw 4.000–15.000 (PLGA), and Tween^®^ 80 were supplied by Merck (Milan, Italy). Ethanol (96% purity) was obtained from J.T.Baker (Deventer, The Netherlands). Acetone and dialysis membrane (molecular weight cut off (Mwco) 3000 Da, diameter 11.5 mm; Spectra/Por^®^) were purchased from VWR International PBI Srl (Milan, Italy). Hydroxypropyl-β-cyclodextrin was obtained from Roquette Freres (Lestrem, France). Deionized water was used for all the preparations.

### 2.2. Preparation of Unloaded Nanoparticles

Nanoprecipitation technique was applied to prepare PLA (NPA) and PLGA (NPB) NPs with slight modification of a previously reported process [28]. PLA or PLGA polymer (3.6 mg/mL) was dissolved in acetone. The organic phase (5 mL) was poured, drop by drop, into 10 mL of water/ethanol mixture (1:1), containing 0.05% (*w*/*v*) Tween^®^ 80, under magnetic stirring (500 rpm) at room temperature, thus forming a milky colloidal suspension. The organic solvents were removed under vacuum by a rotavapor (Buchi) at 40 °C.

### 2.3. Physico-Chemical Characterization

The particle size (Z-ave) and the polydispersity index (PDI) were determined by photon correlation spectroscopy (PCS). PCS was performed using a Zetasizer Nano ZS90 (Malvern Instruments Ltd., Malvern, England) and the experiments were carried out using a 4 mW He-Na laser beam with a 633 nm wavelength. The following parameters were used for these experiments: temperature 25 °C, medium refractive index 1.330, medium viscosity 1.0 mPa s, and dielectric constant value 80.4. The analysis of a sample consisted of 3 sets of measurements, and the results are expressed as mean size ± standard deviation (SD). Each sample was analyzed into disposable sizing cuvettes (DTS 0012).

Zeta potential (mV) was measured using the same instrument. Electrophoretic mobility for each sample was revealed at 25 °C, using the Smoluchowski constant with a value of 1.5 to obtain the corresponding ZP values.

### 2.4. Osmolarity and pH

The osmolarity of NPs was analyzed by freezing point depression (FPD) using a digital osmometer (Osmomat 030, Gonotec, Berlin, Germany) and as calibration solutions distilled water and sodium chloride 0.9%. The value reported for each sample is the mean of 3 different measurements. The determination of pH was carried out using a pH-meter at 25 °C (Checket, Hanna Instrument, Woonsocket, RI, USA) which was calibrated before each use with 3 buffer solutions at pH 4.01 ± 0.02; 7.00 ± 0.02 and 10.00 ± 0.02. Three measurements were made for each sample.

### 2.5. In Vitro Cytotoxicity Test of Unloaded Nanoparticles

#### 2.5.1. Cell Cultures

Primary cultures of microvascular pericytes were obtained from bovine retinas as already described [29]. Briefly, the cells were homogenized and filtered through a nylon filter (80 μm). Phosphate-buffered saline (PBS) at pH 7.4 was supplemented with collagenase-dispase and bovine serum albumin, at concentrations of 1 mg/mL and 0.5%, respectively. The micro-vessels were immersed in the PBS solution for 20 min and maintained at 37 °C. The homogenate was centrifuged for 10 min at 1000× *g*. The isolated cells were plated in Dulbecco’s Modified Essential Medium (DMEM) supplemented with 20% fetal bovine serum (FBS), 2 mM glutamine, 100 U/mL penicillin and 100 µg/mL streptomycin. Culture plates were previously covered with a thin layer of gelatin. At confluence, the cells were trypsinized and seeded in new petri dishes in DMEM at 10% fetal bovine serum.

Bovine microvascular endothelial cells (BMVEC) were purchased from Sigma (Milan, Italy) and fed with Ham’s F10 medium supplemented with 10% FBS, 80 µg/mL heparin, 2 mM glutamine, 100 U/mL penicillin, and 100 µg/mL streptomycin. All experiments were carried out using cells at passage 3–4.

#### 2.5.2. MTT Assay

Pericyte and endothelial cells were seeded in 96-well plates at a cell density of 1.5 × 10^4^ per well. 3-[4,5-dimethylthiazol-2-yl]-2,5-diphenyl tetrasodium bromide (MTT) (Chemicon, Temecula, CA, USA) was used to perform the cell viability assays. Prior to treatment, cells were incubated at a temperature of 37 °C overnight and then treated for 24 h and 48 h in the absence (control) or the presence of NPA and NPB (0.25–5 mg/mL). After incubation periods, 10 μL of MTT reagent (5 mg/mL) was added to each well and the cells were incubated at 37 °C for a further 3 h. Formazan crystals were solubilized under constant agitation with 100 µL of DMSO for 10 min. The absorbance was detected at a wavelength of 570 nm with plate reader (Synergy 2-bioTek). All experiments were performed at least 6 times in triplicate.

### 2.6. FA-Loaded Nanoparticles

FA-loaded NPs were obtained with the same procedure described in Section 2.2. The active compound (1% *wt/wt*, drug/polymer) was added to the organic phase and the preparation proceeded as described above [30,31].

### 2.7. Purification Steps

Nanosystems were subjected to purification by two methods: dialysis and centrifugation, with the aim of removing any residual surfactant or unloaded drugs. The removal of unstructured polymer chains in the nanocarriers was not considered, as their molecular weight is higher than the cut-off of that the dialysis membrane used. In order to observe any physico-chemical properties variation due to purification processes, the NPs suspensions were monitored in terms of mean size, PDI, and surface charge, before and after the purification phases. Centrifugation was performed with a Thermo-scientific SL 16R Centrifuge (Thermo Scientific Scientific Inc., Waltham, MA, USA) at 15,777× *g* for 1 h at 8 °C. The obtained supernatants were collected for high performance liquid chromatography (HPLC) analysis, pellets were resuspended in water and characterized through PCS analysis. For dialysis, previously hydrated cellulose membranes (Mwco 3000 Da, diameter 11.5 mm; Spectra/Por^®^) were used. Membranes containing the colloidal suspensions were immersed in 500 mL of distilled water. Dialysis of each sample (NPA-FA and NPB-FA) was performed with different frequencies of water changes per hour (L/h). In the first case equal to 0.5 L/h (2.5 L in 5 h with 5 water changes) and in the second equal to 1 L/h (3 L in 3 h with 6 water changes). Dialyzed samples were collected and centrifuged at 15,777× *g* for 1 h at 8 °C; the obtained supernatants were then analyzed by HPLC, pellets were resuspended and subjected to PCS analysis.

### 2.8. Encapsulation Efficiency

The percentage of the encapsulated FA into the polymeric matrix of NPs was determined both after centrifugation and after dialysis performed with frequency of water changes of 0.5 and 1 L/h. Samples, including those purified by dialysis, were centrifuged in order to obtain separation of pellet from supernatant. The obtained supernatants were analyzed by HPLC to evaluate the drug concentration; each amount of the sample was quantified by measuring the UV absorbance at 320 nm. The EE was calculated by the difference between the amount of drug entrapped inside the NPs and the total quantity of drug employed to prepare the nanosystems, according the following equation [32]:

$$\mathrm{EE}=\frac{{\mu g\;\mathrm{FA}}_{\mathrm{tot}}-\mu g\;\mathrm{FA}\;\mathrm{in}\;\mathrm{supernatant}}{{\mu g\;\mathrm{FA}}_{\mathrm{tot}}}\times 100$$

### 2.9. Yield of Purification Process

The dialysis purification yield was calculated to select the most efficient method to remove unencapsulated FA from the systems. Purification efficiency was expressed as the percentage amount of dialyzed FA compared with the unencapsulated amount. Dialyzed samples were collected and centrifuged. The concentration of FA in the obtained supernatants was quantified by HPLC analysis, by measuring UV absorbance at 320 nm. The percentage of purification was calculated using the following equation:

$$\mathrm{Purification}\;\mathrm{efficacy}\;\left(\%\right)=\frac{\mu g\;\mathrm{FA}\;\mathrm{in}\;\mathrm{supernatant}}{\left({\mu g\;\mathrm{FA}}_{\mathrm{tot}}-\;\mu g\;\mathrm{encapsulated}\;\mathrm{FA}\right)}\times 100$$

Each experiment was performed in triplicate and the results represent the mean ± SD.

### 2.10. Stability Study of Resuspended Cryoprotected Freeze-Dried Formulations

The suspensions of purified NPs were mixed in a 1:1 ratio with 10% (*w*/*v*) of HP-β-Cyd to achieve a final cryoprotectant concentration of 5% (*w*/*v*). The resulting formulations were frozen and freeze-dried for 24 h (Freeze Dryer Edwards Modulyo, Akribis Scientific Limited, Knutsford, Cheshire, UK). The resuspended cryoprotected freeze-dried NPs were analyzed to evaluate potential changes over time of Z-Ave, PDI, ZP, osmolarity, and pH. The analyses were conducted on the NPs lyophilized powder, resuspended with the same volume of water lost during the drying phase [33]. After reconstitution, the above parameters were analyzed (zero time), after that, all of the formulations were stored in the refrigerator (5 °C) and tests were run again after 7, 14, 21, and 28 days.

### 2.11. In Vitro Release Profile of FA-Loaded NPs

The in vitro drug diffusion profiles of non-encapsulated FA solution (in PBS, pH 7.4) and the release profiles of drug-loaded NPs (NPA-FA and NPB-FA) were evaluated. The amount of FA released from NPs was measured after centrifugation of the samples, performed at 15,777× *g* rpm at 8 °C for 1 h; the obtained supernatants were subjected to HPLC analysis, the pellets were resuspended in a 5% (*w*/*v*) of HP-β-Cyd solution and freeze-dried. Lyophilized NPs were resuspended in 1 mL of PBS pH 7.4 [34]. The suspensions were placed into a cellulose membrane dialysis tubing (Mwco 3.5 kDa, flat width 18 mm, diameter 11.5 mm; Spectra/Por^®^ Dialysis Membrane) and incubated in 19 mL of medium (PBS, pH 7.4), which was maintained under magnetic stirring at 37 °C, up to 48 h. Release medium (500 µL) was sampled at predetermined time points (0, 1, 2, 3, 4, 5, 6, 7, 8, 24, and 48 h) and immediately replaced with the same volume of fresh medium, to maintain the sink condition. FA concentration in the collected samples was quantified by HPLC analysis. Release study was performed in triplicate for each formulation. The release curve was drawn according to the average and SD of 3 values at each moment.

### 2.12. HPLC Analysis

HPLC analysis was performed at room temperature using a 1050 Hewlett-Packard instrument (Hewlett-Packard, Milan, Italy) equipped with a 20 μL injection valve Rheodyne 7125 (Rheodyne Inc., Cotati, CA, USA) and a UV-VIS detector (Hewlett-Packard, Milan, Italy). Mobile phase consisted of a mixture of 81:19 (*v*/*v*) acetonitrile: acetic acid (2% *v*/*v*). Stationary phase was a 4.6 × 15 cm C 18 column (Waters, Milan, Italy). Effluent was monitored at a wavelength of 320 nm, with a flow rate of 1 mL/min. The standard calibration curves were prepared at different dilutions of FA in methanol. The linear regression coefficient determined in the range 0.05–10 μg/mL was 0.9997. No interference resulting from other components was observed.

### 2.13. Scanning Electron Microscopy (SEM)

NPs morphology was assessed by SEM study. The samples were prepared for the electron microscope with a spin-coating procedure at 500 rpm for 1 min with a Suss Microtech instrument and left to dry in air for a few hours. To ensure good conductivity, all of the samples were then coated with 5 nm of gold sputtering at a pressure of 10^−3^ mbar with an Emitech K500X equipment. The SEM were acquired at a low voltage of 3 KV with an InLens detector by using a Field Gemini microscope from Zeiss.

### 2.14. Thermal Analysis of Unloaded and FA-Loaded Cryoprotected Freeze-Dried Nanosuspensions

A DSC1 Star System apparatus (Mettler Toledo, Schwerzenbach, Switzerland) was used to perform calorimetric analyses. The DSC detection system consisted of a Mettler Full Range ceramic sensor (FRS5) with 56 thermocouples and a high sensitivity sensor (HSS8) with 120 thermocouples. The signal time constants were respectively equal to 1.8 and 3.1 s, while the digital resolution of the measurement signal was 16.8 million points. The sampling rate was maximum 50 values/s. Calorimetric resolution and sensitivity of FRS5 and HSS8 sensors, determined through the TAWN test, were respectively between 0.12–0.20 and 11.9–56.0. Each DSC scan had an accuracy of ±0.2 K, a precision of ±0.02 K, and a resolution of ±0.00006 K. Optiplex 3020 software at Mettler Star^®^ Dell was used for the data acquisition. DSC aluminum pans (40 μL) were filled with pure FA, pure polymers, cryoprotectant, cryoprotected freeze-dried empty NPs (NPA and NPB), as well as loaded with FA (NPA-FA and NPB-FA) before sealing. All samples were submitted to heating and cooling cycles in the temperature range 20–200 °C at a scanning rate of 5 °C/min (heating) and 10 °C/min (cooling).

### 2.15. FT-IR Spectroscopy Measurements

Pure FA, pure polymers, cryoprotectant, cryoprotected freeze-dried empty NPs (NPA and NPB) and loaded with FA (NPA-FA and NPB-FA) were analyzed using FT-IR spectrophotometer (Perkin-Elmer Spectrum RX I, Waltham, MA, USA). The instrument was equipped with an attenuated total reflectance (ATR) accessory and a diamond window and zinc selenide crystal (diamond/ZnSe). The dried samples were mixed with potassium bromide (KBr anhydrous of FT-IR grade) to obtain a homogeneous mixture, which was compressed into 1 mm pellets. The background was acquired from pure KBr pellet. For each sample, 20 scans were collected over the range of 400–4000 cm^−1^ at a resolution of 2 cm^−1^ at room temperature.

### 2.16. Statistical Analysis

All results are reported as mean ± SD. The results were analyzed using one-way ANOVA followed by Tukey–Kramer multiple comparisons test; differences between groups were considered significant for a *p*-value <0.05. The *t*-test was used to calculate the statistical significance in the MTT assay; the percentages obtained relative to the control group were considered not significant for *p* > 0.05, significant for *p* < 0.05, very significant for *p* < 0.01 and extremely significant for *p* < 0.001.

## 3. Results and Discussion

In the present study, NPs have been produced by a solvent displacement technique. Tween 80, a non-ionic surfactant, was added in order to reduce the dynamic interfacial tension and to increase the steric repulsion between NPs [28]. Its non-ionic nature allows it to be included in the ophthalmic formulations, but this is acceptable since it does not induce strong eye irritation [35]. The concentration chosen for the emulsifier (0.05% *w*/*v*) was selected because it is considered a suitable amount both for obtaining small diameter particles and for the demonstrated ocular safety and tolerability [28,36].

### 3.1. Influence of Unloaded NPs Concentration on Cell Viability of Primary Cultures of Micro-Capillaries Pericytes and Endothelial Cells

An NPs system proposed for ocular administration must be able to deliver the active agent without compromising the viability of the host cells. To assess if NPA or NPB could induce cytotoxicity, MTT bioassay was performed on primary cultures of micro-capillaries pericytes and endothelial cells. Unloaded NPs were studied at different concentrations (0.25–5 mg/mL) to evaluate the effect on cell viability and the potential application of these systems as FA nanocarriers for ocular therapy.

**Figure 1:**
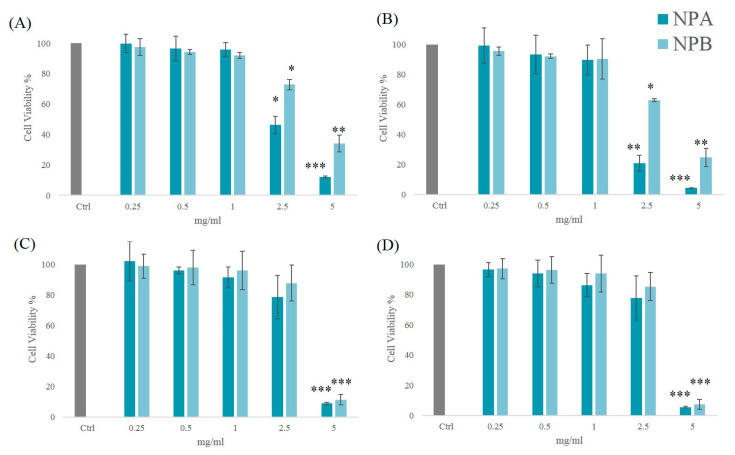
Cytotoxicity of NPA and NPB NPs on primary endothelial cells after (**A**) 24 h and (**B**) 48 h of incubation and on primary retinal pericytes cells after (**C**) 24 h and (**D**) 48 h of incubation at different concentrations (5; 2.5; 1; 0.5; 0.25 mg/mL). Three independent experiments were performed in sixfold. Error bars depict the S.D. of the mean. *t*-test was used to calculate statistical significance of the percentages obtained versus control group. [ns = not significant (*p* > 0.05); * = significant (*p* < 0.05); ** = very significant (*p* < 0.01); *** = extremely significant (*p* < 0.001)].

Figure 1 shows cell viability vs NPs concentration (mg/mL). An important consideration is that cell viability strictly depended on the type of cell line as well as on the concentration tested. The results obtained from the analysis of NPA and NPB carriers on BMVEC were plotted as a function of the incubation time, which is equal to 24 (Figure 1A) and 48 h (Figure 1B). Regarding the data obtained at 24 h, the cells incubated with NPA and NPB showed a high viability (>90%) in the concentration range 0.25–1 mg/mL. Viability at 48 h follows the same trend, with no significant reduction. The evidence of a more marked reduction is observable for NPA at higher concentrations. From the statistical analysis of the data, it emerged that the decrease in viability compared to the control recorded for NPA at 2.5 mg/mL oscillates between significant (24 h) and very significant (48 h), it is instead extremely significant at 5 mg/mL. For NPB, the amount of reduction was significant at 2.5 mg/mL and very significant at 5 mg/mL. Therefore, although NPB in the safe range 0.25–1 mg/mL have the lowest cell viability rates, at higher concentrations, they show a less pronounced, although still toxic, reduction. Pursuant to ISO 10993-5, percentages of cell viability above 70% are considered an absence of cytotoxicity [37]. The results obtained showed that the highest concentrations (2.5–5 mg/mL), for both samples and times examined were cytotoxic, resulting in a reduction in viability >30%.

The analysis of NPs on RMP incubated for 24 h showed the absence of toxicity in the concentration range 0.25–2.5 mg/mL for both systems studied (Figure 1C). NPB showed higher viability percentages on the concerned cell line with respect to the NPA. Similar data with the same safety interval were obtained also for cells incubated for 48 h (Figure 1D). Analysis revealed that both formulations at the highest concentration (5 mg/mL) were cytotoxic, resulting in an extremely significant reduction in cell viability.

From the results obtained, it was observed that NPA and NPB showed similar behavior on both cell lines. In detail, absence of cytotoxicity was observed in pericyte cell lines with a wider concentration range (0.25–2.5 mg/mL) than in endothelial cells (0.25–1 mg/mL).

Endothelial cells and pericytes are essential components of the microvessel wall. Pericytes play several roles in the retinal vascular system, from controlling flow to maintaining microcirculation integrity [38,39]. Pericytes work co-dependently with endothelial cells, to which they also provide mechanical support. Among the activities that pericytes regulate are the proliferation and migration of endothelial cells, as well as the production of cytokines for the immune response [40,41,42].

In vivo, therefore, contact and interactions between pericytes and endothelial cells act on different levels of control. Similar results were reported in a study conducted on human pericytes and endothelial cells, where differences in cell lines cultured solitary and in co-culture were observed. The results showed that DNA synthesis of endothelial cells in single culture was reduced by 30% compared to cells co-cultured with pericytes. Therefore, in vitro co-culture studies should be more reliable and predictive in the evaluation of biological cellular responses [43].

### 3.2. Influence of the Purification Process on Physico-Chemical Properties of Nanocarriers

As shown by the physico-chemical characterization of formulated systems (Table 1), the particle size ranged between 158 and 219 nm, thus, NPs were obtained [44,45]. In particular, the mean particle size of unloaded NPs was ~158–170 nm and of FA-loaded NPs was ~178–219 nm. The particle size distribution was very narrow in all cases (PDI less than 0.3), corresponding to monodispersed systems [46].

The ZP of NPs was strongly negative, ranging between −23.8 ± 2.22 to −39.0 ± 1.40 mV. The negative ZP values could be attributed to the presence of terminal carboxylic groups of the polymers, which confer to the matrix of a negative surface charge [28,47,48].

The ZP value showed a reduction of 6 mV in absolute value when the drug was incorporated into the systems, probably due to its precipitation on the surface on NPs. The selection of the organic solvent and its evaporation played a crucial role in this process. Acetone can diffuse into the continuous phase and temporarily increase the drug solubility. As a result, when the organic solvent was completely evaporated, FA could precipitate and deposit onto the NP surface, masking their surface charge [49]. The results of osmolarity values of the obtained formulations showed the achievement of isoosmolar systems with the tear fluid and pH values of 7.3, which fall within the ocular tolerability range.

**Figure 2:**
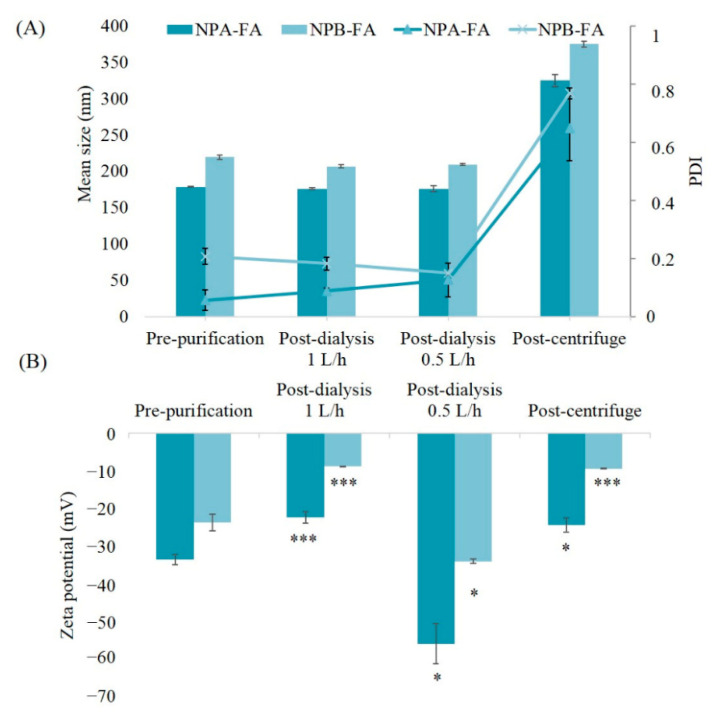
Mean size, PDI (**A**) and zeta potential (**B**) of the samples NPA-FA and NPB-FA. *t*-test was used to calculate statistical significance of the percentages obtained versus control group. [ns = not significant (*p* > 0.05); * = significant (*p* < 0.05); *** = extremely significant (*p* < 0.001)].

In order to evaluate the influence of purification methods on the physico-chemical properties of the obtained systems and to select the appropriate process for these nanocarriers, we characterized NPA-FA and NPB-FA before and after the purification processes (Figure 2). The results obtained showed that both formulations subjected to the centrifugation process endured an increase in mean size, passing from 178.6 to 325 nm for NPA-FA and from 219.3 to 357.2 nm for NPB-FA. This increase should be attributed to the speed used during the centrifugation, which is able to generate collision forces between NPs. The mechanical induced stress leads to the formation of non-redispersible aggregates, according to Sari et al. [50]. After centrifugation the samples showed an increase in PDI reaching values of 0.649 and 0.769 respectively for NPA-FA and NPB-FA. The increase in polydispersion confirmed that high speed used during the centrifugation process caused the formation of aggregates. ZP of both centrifuged NPs showed a reduction of about ten mV. This decrease is a result of the aggregation phenomena that cannot keep the surface properties of NPs unaltered, probably due to the reduction in the total surface area [51].

No significant variation was highlighted in the average dimensions of NPs which remained rather constant after the dialysis process. Systems obtained after dialysis maintained a low PDI value (<0.2).

For dialyzed samples, significant changes on ZP (*p*-value <0.05) were observed based on the volumes of water exchanged per hour. As reported in Figure 2, a reduction in ZP values can be observed for samples dialyzed against 1 L/h. This result could be due to the further adsorption of the unloaded drug onto the surface of NPs. Moreover, the higher frequency of water exchange avoids the formation of possible surfactant micelles that could entrap the free drug and prevent its diffusion through the membrane. The ZP values of the samples dialyzed against 0.5 L/h of water showed an increase in absolute value of this parameter. The reason for this could be an increase in the osmotic pressure in the dialysis solution. It has been shown that the osmotic pressure of a non-ionic aqueous surfactant solution in the micellar region increases with increasing concentration of the surfactant [52]. In our case, lower frequency of water exchanges (0.5 L/h) may lead to increased surfactant concentration in the dialysis medium forming micelle. The formation of micelle could sequestrate a fraction of the unloaded drug. The difference in osmotic pressure that was generated in the dialysis medium could hinder the progressive diffusion of the surfactant molecules from the nanodispersion, preventing proper dialysis of the samples [53].

In fact, to support this hypothesis and to evaluate the influence of frequency of water exchanges on dialysis efficacy, a comparison of obtained data was made (Table 2). The results showed that the dialysis technique, which allows a better purification yield of drug (>50%) and is able to remove the greater percentage of FA, is that performed with a frequency of water changes equal to 1 L/h. Samples dialyzed against 0.5 L/h of water showed lower purification efficiency. The reason for this could be a sequestration of the drug from the surfactant micelles present in dialysis medium [54]. In regard to the reasons for the major purification of NPB-FA, this could be attributed to the lipophilic nature of drug. Consequently, the fact that FA is less related to the PLGA polymer, which has hydrophilic groups in its structure, is retained less from NPB-FA [55]. The poor affinity of drug for this type of polymer could reduce its solubilization inside the matrix, therefore a higher amount of drug could remain adsorbed on the NPB-FA surface. Thanks to the ease with which FA is removed from the surface of PLGA based NPs, a higher dialysis percentage may have been obtained [56].

### 3.3. Encapsulation Efficiency and In Vitro Release Profile of FA-Loaded Nanocarriers

The entrapment efficiency of FA (1% *wt*/*wt*) in the NPs prepared by the nanoprecipitation method was calculated for both purification methods investigated. The results are shown in Table 3. The EE obtained for centrifuged systems ranged from 64.86 to 75.16%, respectively for NPB-FA and NPA-FA. For dialyzed samples, the percentages showed higher values ranging from 81 to 90% and it was observed that by subtracting the non-dialyzed amounts of drug (Table 2), the results were identical to those obtained for the samples purified by centrifugation. Therefore, the efficiency obtained for the dialyzed samples was defined as ‘apparent EE’, consisting of the encapsulated drug, plus the amount of FA not removed by dialysis.

It was demonstrated that the encapsulation yield depends on several factors, such as: the solvent miscibility in the aqueous phase, the precipitation speed rate which leads to polymer solidification, and the drug solubility into the polymer used [57].

The values obtained could be related to the high solvent miscibility in the continuous phase. It has been shown that if the solvent has a good miscibility, such as in acetone, a very fast solidification of the polymer may occur during the evaporation step. Especially for a hydrophobic drug, a rapid solidification is advantageous in order to obtain high EE, because the dense polymeric shell that is obtained acts as a diffusion barrier for the drug [54]. Additionally, faster hardening can be observed when the volume ratio of continuous to dispersed phase increased (as in our case where a 2:1 ratio is used), which could result in fast solidification of the systems and so improve the effectiveness of encapsulation [58].

NPB-FA showed a lower drug loading than NPA-FA made up of PLA polymer. The lower EE could be attributed to the lower ability of a drug interaction with the polymer. This capacity depends on the drug interaction with the matrix, therefore the more of the drug that is akin to the polymer, the greater the amount of encapsulated FA will be [59].

PLA polymer has a greater hydrophobicity with respect to PLGA, which instead contain a glycol portion in its structure, which provides hydrophilic properties to the polymer [49].

**Figure 3:**
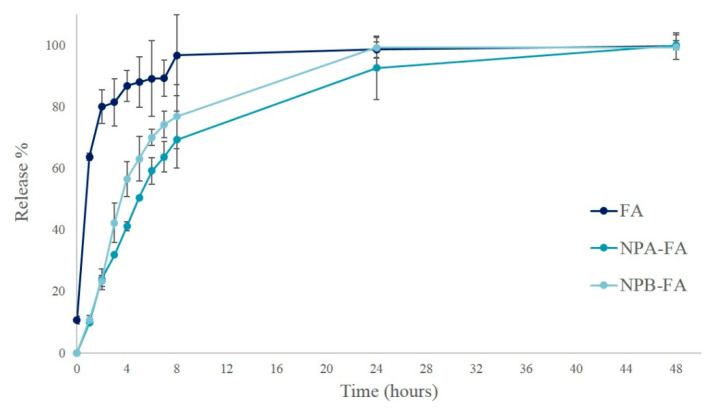
In vitro release profiles of pure drug, NPA-FA and NPB-FA in phosphate buffered solution (pH 7.4) at 37 °C.

Release curves of pure drug and FA-loaded NPs of are shown in Figure 3. More than 80% of FA powder released quickly in 2 h under PBS conditions (pH = 7.4). The release rate was similar to a straight line between 0 and 2 h, then the slope tends to decrease. After 8 h, the drug concentration was almost unchanged, suggesting that FA was completely released into the solution.

As shown in the graph, the slope in the NPs release curves is reduced compared to the pure drug, indicating that the encapsulation of the drug was able to provide a more prolonged release over time. FA release from the NPs in the first 2 h was about 23% for both systems. This premature release could be due to the amount of unloaded FA that was not removed through dialysis. The release profile then increased gradually, reaching about 50% after 4 h from NPB-FA and 5 h from NPA-FA. The FA concentration increased to the theoretical maximum concentration in 48 and 24 h for NPA-FA and NPB-FA, respectively.

Although both curves show similar trends, NPA-FA releases lower drug concentrations over time than NPB-FA. The release profile of a drug is governed by its partitioning between the polymer matrix and the aqueous release medium [60]. The difference in release rates could therefore be attributed to the nature of the investigated matrices. NPA-FA, which has demonstrated higher EE, showed a relatively lower drug release than NPB-FA containing a hydrophilic portion in the matrix composition. A similar behavior was observed in a study by Panyam et al., which demonstrated a close correlation between drug release profiles and the degree of hydrophilia of the polymer matrix [49]. The release process is controlled by the degradation rate of the polymer [61,62]. During this process the drug diffuses through the hydrated polymer matrix to the release medium. The water uptake into the systems relaxes the polymer chains and increases the rate of diffusion of drug molecules [63]. Therefore, a higher release rate is justified for NPB-FA particles more hydrophilic than NPA-FA.

### 3.4. Stability Studies on Resuspended Freeze-Dried FA-Loaded NPs

**Figure 4:**
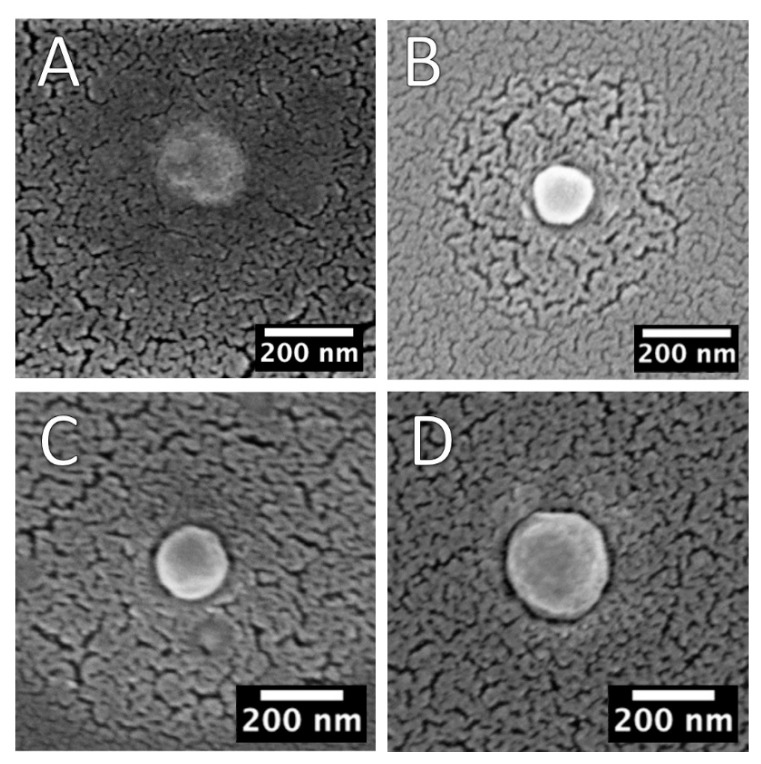
SEM micrographies of: (**A**) NPA (**B**) NPB-FA (**C**) NPB and (**D**) NPB-FA.

Liquid polymeric nanosuspensions have some limitations related to the integrity of the formulations. Among these, a recurring phenomenon is the formation of undesirable degradation products generated by hydrolysis of the polymeric material [64,65]. Another limitation of nanosuspensions has been reported about the possibility of premature release of the encapsulated drug [66]. To exceed the above limits, samples were converted from aqueous suspensions to dried powders through the freeze-drying process. However, the freezing and drying steps for removing water from nanosystems subject them to various stresses, so cryoprotective agents are usually added to the formulation with the aim to preserve the structure and morphology of colloidal systems and increase their stability during storage [67,68]. Carbohydrates are among the most commonly used adjuvants able to prevents aggregation phenomena. A good quality lyophilized product is characterized by quick and easy reconstitution, as well as by maintaining the particle size [69]. In our previous studies it was demonstrated that an elegant cake appearance and a short reconstitution time were achieved when 5% (*w*/*v*) of HP-β-Cyd was employed as cryoprotectant, because, thanks to its cyclic structure, it is able to be easily absorbed onto the NPs surface during the sublimation step, ensuring also an easy reconstitution of the dried material [28,68]. Furthermore, the concentration of cryoprotectant used is able to provide a dispersion of NPs with an adequate tonicity for ocular administration [70]. SEM scans of cryoprotected and freeze-dried empty and FA-loaded NPs are shown in Figure 4. Results showed NPs with a spherical shape, smooth surface, and a reduced average-diameter compared to pre-lyophilisation values, as reported in Table 1.

**Figure 5:**
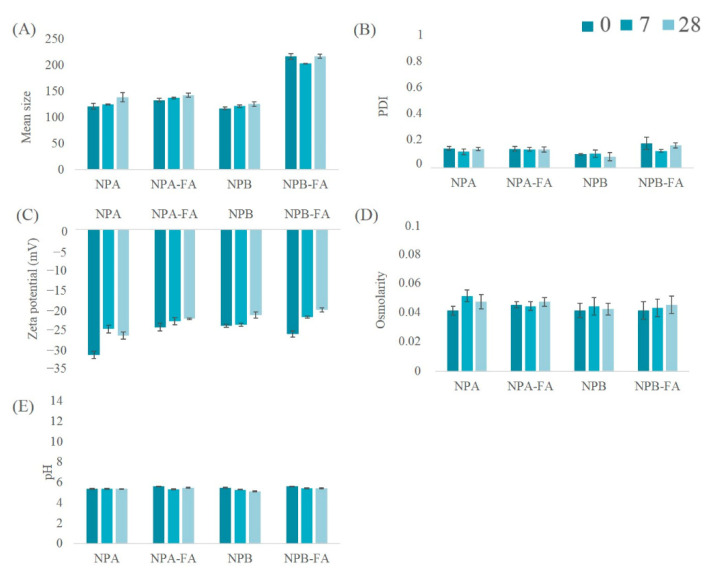
Mean size (**A**) index of polydispersion (**B**) zeta potential (**C**) osmolarity (**D**) and pH (**E**) of the samples stored at 5 °C.

The results obtained from the stability studies conducted on lyophilized systems resuspended with distilled water and stored at refrigeration temperature have shown a chemical-physical stability almost unchanged over time. The data collected are shown in Figure 4. PCS analysis provided further confirmation of the reduction in the mean size of the systems (Figure 5A), which remained stable during the storage time considered. In addition, the PDI of all the analyzed formulations maintained constant values, always below 0.2 (Figure 5B). From the analysis of the ZP (Figure 5C) it is possible to observe a slight decrease in this parameter over the course of 28 days, despite the fact that the surface charge of the systems always remains greater than −20.5 mV. In general, dispersions with high absolute values of ZP are considered stable because the electrical repulsion between the charges of the NPs is able to reduce the aggregation capacity of systems [61,71]. Therefore, NP formulations were found to be physically stable, and aggregation of colloidal particles was probably prevented due to adequate values of ZP. Figure 5D,E show respectively measurements of osmolarity and pH values of the NPs suspensions, whose variations over time have proved to be practically irrelevant.

### 3.5. Thermal and Infrared Analyses of Cryoprotected and Freeze-Dried Nanoparticles

**Figure 6:**
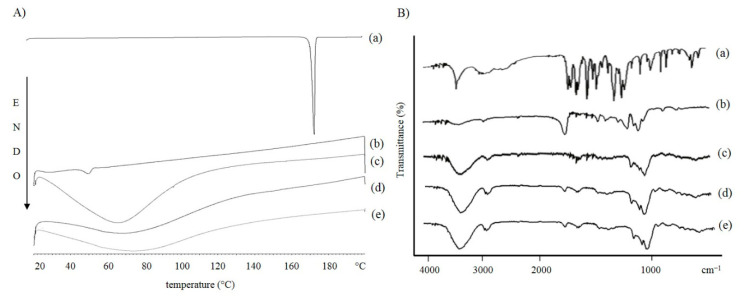
(**A**) DSC and (**B**) FT-IR curves of FA (**a**) PLA polymer (**b**) HP-β-CD (**c**) unloaded NPA (**d**) and FA-loaded NPA nanoparticles (**e**).

**Figure 7:**
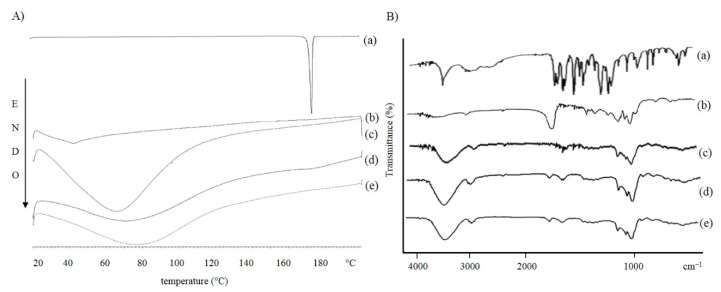
(**A**) DSC and (**B**) FT-IR curves of FA (**a**) PLGA polymer (**b**) HP-β-CD (**c**) unloaded NPB (**d**) and FA-loaded NPB nanoparticles (**e**).

In order to investigate the polymorphic states and crystallinity of the materials used during the preparation and of the cryoprotected and lyophilized formulations, we conducted a DSC study. The NPs made of PLA are represented in Figure 6A, those produced with PLGA are represented in Figure 7A.

As shown in the curves (a) of both graphs, the melting point of FA is 172.3 °C, which represents the characteristic endothermic peak of the drug [72]. The disappearance of the characteristic endothermic FA peak in the thermograms of FA-loaded NPs [Figure 6A and Figure 7A(e)] demonstrates that the drug could be successfully entrapped in the amorphous state within the formulated systems.

As expected, the polymer thermograms show characteristic peaks between 40–50 °C [73]. Regarding the DSC thermograms of the cryoprotected freeze-dried NPs, no obvious melting process occurred. This could be due to the presence of cryoprotectants. It has been shown that cryoprotectant molecules act through water substitution. The stabilization of NPs could be explained as the formation of hydrogen bonds between the polar groups on the polymer surface and the cryoprotectant molecules, resulting in the loss of water [64]. HP-β-CD has a relatively high glass transition temperature (Tg). When it is arranged around the NPs, it changes the collapse temperature of the systems. This results in a shorter primary drying phase during lyophilization. The obtained amorphous structures are characterized by a low aggregation capacity which prevents the formation of agglomerates during the freeze-drying process [74,75].

FT-IR spectroscopic analysis was performed to identify the functional groups of the materials used in the preparation and the chemical interactions that could have occurred in the formulated carriers. The spectra of the raw materials and cryoprotected and freeze-dried NPs, empty and loaded with FA, were scanned. The results are shown in Figure 5B and Figure 6B. The FA showed a peak at 3436 cm^−1^ typical for -OH stretching vibrations, the absorption bands in the range 2968–3016 cm^−1^ corresponded to the presence of the alkane groups. The band at 1690 cm^−1^ was observed for the C=O carbonyl group and the band at 1277 cm^−1^ for the C-O group. The signals at 1619 and 1517 cm^−1^ were related to the vibration of the aromatic ring, while the peak at 1205 cm^−1^ is typical for C-OH stretching and finally, the band at 1035 cm^−1^ for methoxide O-CH_3_ stretching. The polymer spectra also showed characteristic absorption bands. The broad bands at 3400 cm^−1^ are typical for hydroxyl groups, the bands at 2997 cm^−1^ corresponding to the vibration for C-H alkane groups, the characteristic stretching peaks for C=O carbonyl group are shown at 1751 and 1761 cm^−1^ for PLA and PLGA, respectively. The bands between 1300 and 1400 cm^−1^ were characteristic for C-H alkane groups bending vibration and the bands in the region between 1272 and 1048 cm^−1^ were characteristic for C-O vibration. Cryprotectant FT-IR spectrum showed an absorption band at 3420 cm^−1^ for -OH stretching vibration, a peak at 2933 cm^−1^ for alkane group vibration, and a signal at 1157 cm^−1^ for C-O vibration. Cryoprotected and freeze-dried NPs scans, empty and loaded with FA, confirmed the results obtained from the thermal analysis, which showed a similar trend to cryoprotectant. In the infrared spectra of PLA (Figure 5B) and PLGA (Figure 6B) NPs, all of the characteristic peaks of FA disappeared, while the cryoprotectant typical peaks were detected at 3400 cm^−1^ for -OH group, at 2900 cm^−1^ for alkane group and the bands in the region between 1035–1157 cm^−1^ for C-O groups. In addition, a characteristic polymer peak at 1750 cm^−1^ for carbonyl group was detected. An interesting feature of the NPs spectra was the appearance of a peak at 1650 cm^−1^, attributable to the H-O-H bending band, which suggested a possible chemical interaction between the cryoprotectant and the polymer matrix.

Therefore, it could be stated that NPs consist of a polymeric matrix in which the drug was present in a dispersed form and partially exposed on the surface, while the external area of the matrix is covered by a cryoprotective layer capable of maintaining the integrity of the nanosystems.

## 4. Conclusions

In this work, the synthesis of empty PLA (NPA), PLGA (NPB), and polymeric NPs loaded with FA was developed for ophthalmic applications. The obtained systems were characterized by PCS analysis, data show homogeneous particle populations with a PDI <0.2, with adequate dimension for ophthalmic administration and a strongly negative ZP, which reduces the probability of obtaining aggregates.

To obtain suitable formulations for in vitro or in vivo studies, different purification techniques were analyzed. Centrifugation proved to be the least suitable method because it involves heterogeneous and non-redispersible aggregate formation. Dialysis on the other hand, did not affect the dimensional parameters or PDI values for both frequencies of water changes tested. Purification efficacy was also evaluated in terms of drug removed. Dialysis, which allows a high FA purification yield, was performed with a frequency of water changes equal to 1 L/h. The formulations were characterized in terms of osmolarity and pH, making them suitable for ocular administration with well tolerated pH (7.3) and isotonic osmolarity values with the tear fluid between 258–265 mOsm/Kg. The tolerability of the blank carriers was confirmed by cell viability assays. NPA and NPB showed no toxic effect in the concentration range 0.25–1 mg/mL on endothelial cells, while on pericytes, NPs were safe at a higher concentration range of 0.25–2.5 mg/mL. However, further studies would be required to ascertain the toxicity of polymeric carriers in vivo. The encapsulation effectiveness was also assessed for both loaded formulations (NPA-FA, NPB-FA) with drug entrapment yields of 75.16 and 64.86%, respectively. The results of in vitro release studies showed that obtained systems are able to provide a controlled FA release up to 48 h. Using 5% (*w*/*v*) HP-ß-cyclodextrin as cryoprotective agent, the polymeric carrier systems can be freeze-dried, ensuring good physical-chemical properties upon reconstitution and over 28 days. From the results of morphological analysis, the nanoparticles showed a spherical and smooth surface. Thermal and spectroscopic analyses confirmed that the drug was encapsulated within the polymer matrix. Hypothesis made on the in vitro biological tests must be confirmed with further studies which must also be conducted on FA-loaded NPs, although the obtained results may not be correlated with results of the in vivo studies where cellular homeostasis is governed by multiple factors.

## Figures and Tables

**Table 1:** Mean size, PDI, zeta potential (ZP), osmolarity and pH of loaded (NPA-FA, NPB-FA) and unloaded (NPA, NPB) nanoparticles. Data represent mean standard deviation (SD), *n* = 3.

Sample	Mean Size (nm) ± SD	PDI ± SD	ZP (mV) ± SD	Osmolarity ± SD (mOsm/kg)	pH ± SD
**NPA**	170.400 ± 5.781	0.128 ± 0.028	−39.00 ± 1.40	-	-
**NPA-FA**	178.600 ± 0.289	0.056 ± 0.035	−33.70 ± 1.31	258.3 ± 0.023	7.30 ± 0.533
**NPB**	158.700 ± 1.700	0.130 ± 0.023	−29.70 ± 0.90	-	-
**NPB-FA**	219.300 ± 2.751	0.207 ± 0.028	−23.80 ± 2.22	265.6 ± 0.027	7.33 ± 0.495

**Table 2:** Purification efficiency (%) of NPA-FA and NPB-FA referred to the purification processes using the dialysis method performed with frequency of water exchanges of 0.5 and 1 L/h.

Sample	Frequency of Water Changes (L/h)	Purification Efficiency (%) ± SD
**NPA-FA**	1	28.60 ± 0.211
	0.5	24.13 ± 0.015
**NPB-FA**	1	53.29 ± 2.258
	0.5	30.00 ± 0.785

**Table 3:** Encapsulation efficiency (%) of NPA-FA and NPB-FA and apparent encapsulation efficiency (%) of NPA-FA and NPB-FA referred to the purification processes using the dialysis method performed with frequency of water exchanges of 0.5 and 1 L/h.

Sample	Encapsulation Efficiency (%) ± SD	Frequency of Water Changes (L/h)	Apparent Encapsulation Efficiency (%) ± SD
**NPA-FA**	75.16 ± 5.148	1	89.36 ± 0.085
		0.5	90.22 ± 0.007
**NPB-FA**	64.86 ± 6.357	1	81.27 ± 0.792
		0.5	89.46 ± 0.276

## Data Availability

The data presented in this study are available on request from the corresponding author.
